# Author Correction: Pancreatic Ppy-expressing γ-cells display mixed phenotypic traits and the adaptive plasticity to engage insulin production

**DOI:** 10.1038/s41467-021-26062-9

**Published:** 2021-09-27

**Authors:** Marta Perez-Frances, Léon van Gurp, Maria Valentina Abate, Valentina Cigliola, Kenichiro Furuyama, Eva Bru-Tari, Daniel Oropeza, Taïna Carreaux, Yoshio Fujitani, Fabrizio Thorel, Pedro L. Herrera

**Affiliations:** 1 Department of Genetic Medicine & Development, iGE3 and Centre facultaire du diabète, Faculty of Medicine, University of Geneva, Geneva, Switzerland; 2 Center for iPS Cell Research and Application (CiRA), Kyoto University, Kyoto, Japan; 3 Lab. of Developmental Biology & Metabolism, Institute for Molecular & Cellular Regulation, Gunma University, Maebashi, Gunma Japan; 4 Present Address: Department of Cell Biology, Duke University Medical Center, Durham, NC USA; 5 Present Address: Regeneration Next, Duke University, Durham, NC USA

**Keywords:** Cell biology, Diabetes, Islets of Langerhans, Diabetes

Correction to: *Nature Communications* 10.1038/s41467-021-24788-0, published online 22 July 2021.

The original version of this Article contained an error in the Acknowledgments, which omitted the grant numbers relating to the Swiss National Science Foundation and the European Research Council given for P.L.H. The original version read ‘This work was funded with grants from Swiss National Science Foundation, European Research Council, and Fondation Aclon to P.L.H’. The correct version replaces this sentence with ‘This work was funded with grants from the Swiss National Science Foundation (#310030_192496), the Fondation Aclon and the European Research Council (“Merlin”, #884449) to P.L.H’. This has been corrected in both the PDF and HTML versions of the Article.

