# Supplementary material for: Pancreatic Ppy-expressing γ-cells display mixed phenotypic traits and the adaptive plasticity to engage insulin production
Source: Nat Commun. 2021 Jul 22;12:4458. doi: 10.1038/s41467-021-24788-0 (PMC8298494; doi:10.1038/s41467-021-24788-0)
Supplement: Supplementary file 1 — Supplementary Information [file 41467_2021_24788_MOESM1_ESM.pdf]

# Pancreatic Ppy-expressing γ-cells display mixed phenotypic traits and have adaptive plasticity to engage insulin production

Marta Perez-Frances[1], Léon van Gurp[1], Maria Valentina Abate[1], Valentina Cigliola[1,2], Kenichiro Furuyama[1,3], Eva Bru-Tari[1], Daniel Oropeza[1], Taïna Carreaux[1], Yoshio Fujitani[4], Fabrizio Thorel[1] and Pedro L. Herrera[1,*]

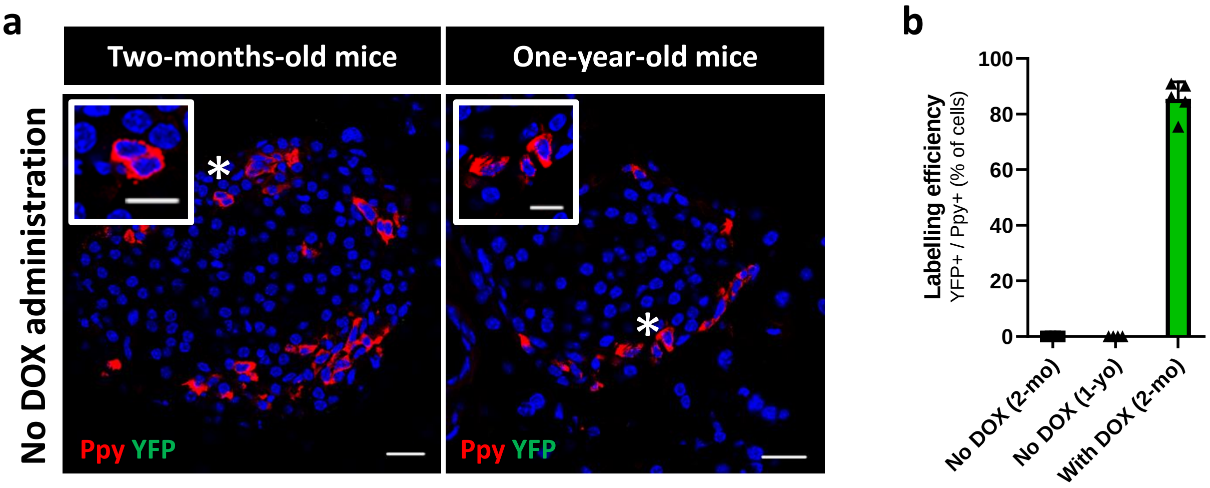


Supplementary Figure 1. **Inducible Ppy-YFPi lineage-tracing. a,** No YFP labelling is observed in untreated two-month-old and one-year-old mice. Immunofluorescence: Ppy, red; YFP, green. Scale bars: 20µm (10µm in insets). **b,** Percentage of Ppy-expressing cells labelled with YFP with or without DOX treatment. No DOX (2-month-old mice): 0,0 ± 0,0; n=4, 1458 $Ppy^+$ cells scored. No DOX (1-year-old mice): 0,0 ± 0,0; n=4, 696 $Ppy^+$ cells scored. With DOX treatment (2-month-old mice): 84,9 ± 0,0; n=8, 5411 $Ppy^+YFP^+$ out of the 6389 $Ppy^+$ cells scored. Error bars are s.e.m. Region of the pancreas: Ventral. Source data are provided as Source Data file (table a, b).

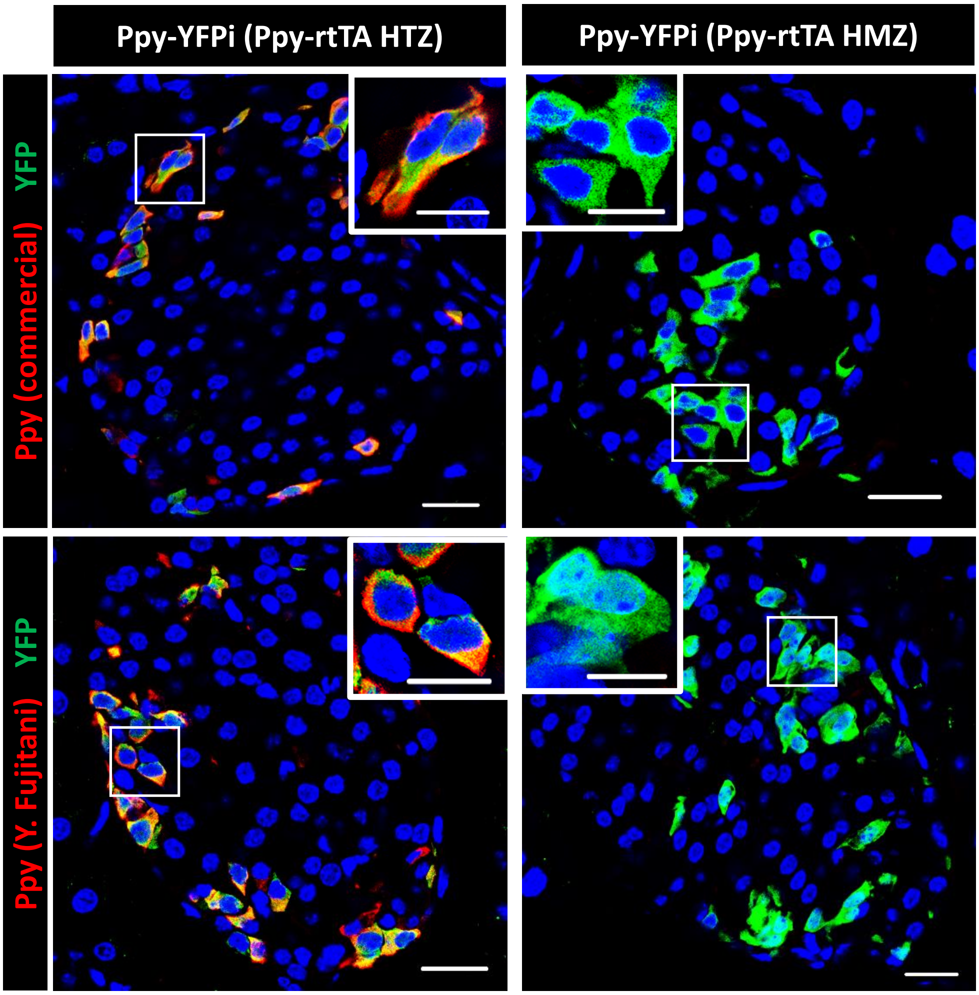


Supplementary Figure 2. **Validation of the anti-Ppy antibodies.** Representative islet from a 2-month-old DOX-treated Ppy-YFPi mouse bearing Ppy-rtTA transgene in heterozygous (HTZ) or homozygous (HMZ) state. Ppy staining (using both anti-Ppy antibodies, red) is lost in YFP (green)-labelled cells from Ppy-rtTA HMZ mice. Any of the YFP-labelled cell in DOX-treated Ppy-rtTA HMZ mice is stained with Ppy hormone. Scale bars: 20μm (10μm in insets). Region of the pancreas: Ventral.

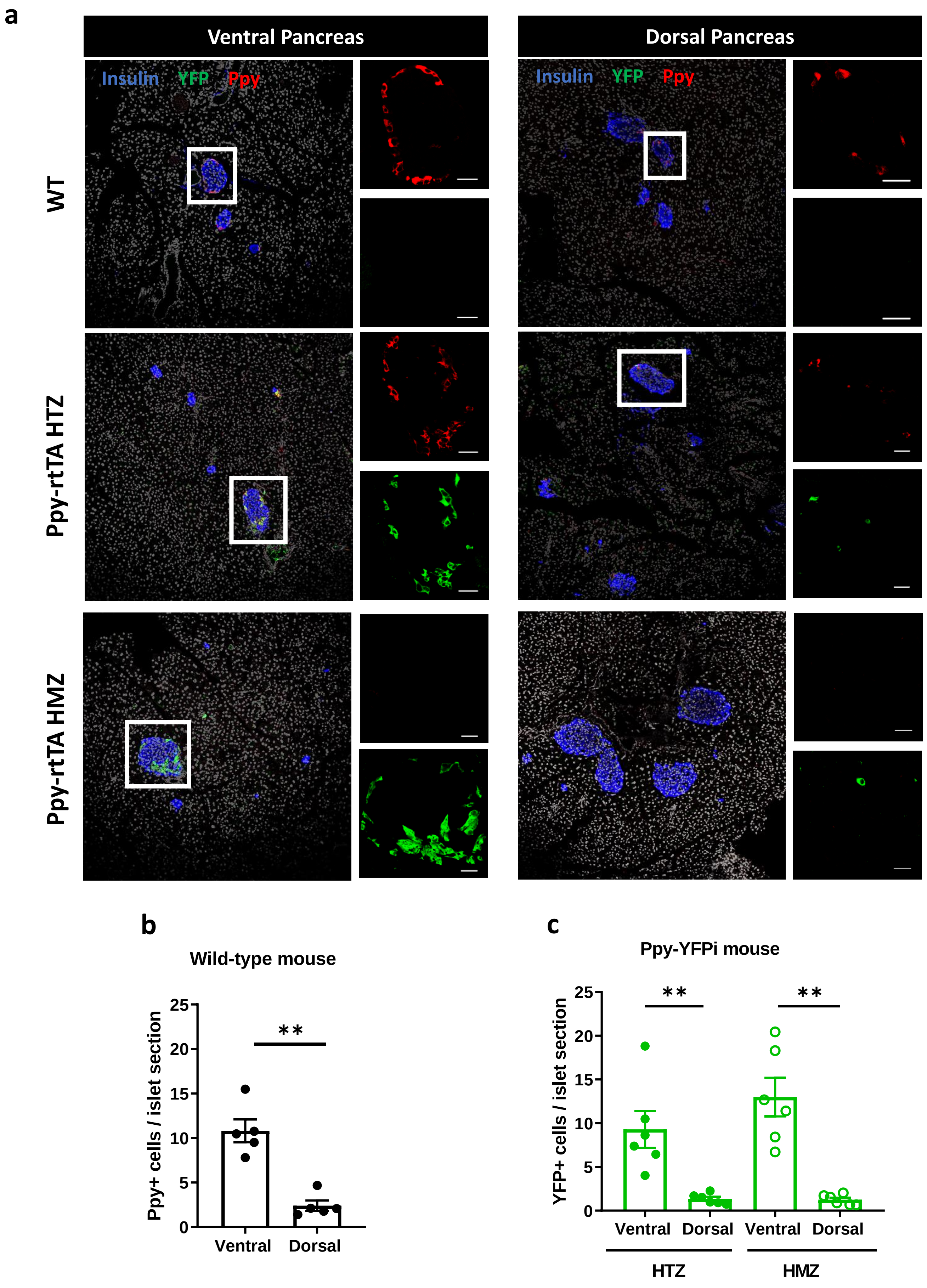

a
Ventral Pancreas
Dorsal Pancreas
Insulin YFP Ppy
Insulin YFP Ppy
WT
Ppy-rtTA HTZ
Ppy-rtTA HMZ
b
Wild-type mouse
Ppy+ cells / islet section
25
20
15
10
5
0
**
Ventral
Dorsal
c
Ppy-YFPi mouse
YFP+ cells / islet section
25
20
15
10
5
0
**
**
Ventral
Dorsal
Ventral
Dorsal
HTZ
HMZ

Supplementary Figure 3. **γ-cell distribution in the pancreas of wild-type and Ppy-YFPi mice.** **a,** YFP-traced γ-cells in Ppy-rtTA heterozygous (HTZ) and homozygous (HMZ) mice follow the same distribution pattern than wild-type mice, being predominantly located in the ventral region of the pancreas. Comparative immunofluorescence of ventral and dorsal pancreatic sections: Ppy, red; YFP, green; Insulin, blue. Scale bar: 40μm (section) or 20μm (islet). **b-c,** Quantification of $Ppy^+$ **(b)** or $YFP^+$ **(c)** cells per islet section in ventral and dorsal sections of wild-type and Ppy-rtTA HTZ and HMZ, respectively. WT mice (n=5 mice, 5768 and 1288 Ppy+ cells scored in ventral and dorsal regions, respectively), Ppy-rtTA HTZ (n=6 mice, 3482 and 1017 YFP+ cells scored in ventral and dorsal regions, respectively) and Ppy-rtTA HMZ (n=6 mice, 3731 and 428 YFP+ cells scored in ventral and dorsal regions, respectively). Data is presented as mean values ± s.e.m. Two-tailed Mann-Whitney test (ns p>0.05; * p≤0.05; ** p≤0.01; *** p≤0.001); wild-type ventral versus dorsal: *P* value = 0.0079, Ppy-YFPi (HTZ) ventral versus dorsal: *P* value = 0.0022, Ppy-YFPi (HMZ) ventral versus dorsal: *P* value = 0.0022. Source data are provided as Source Data file (table c).

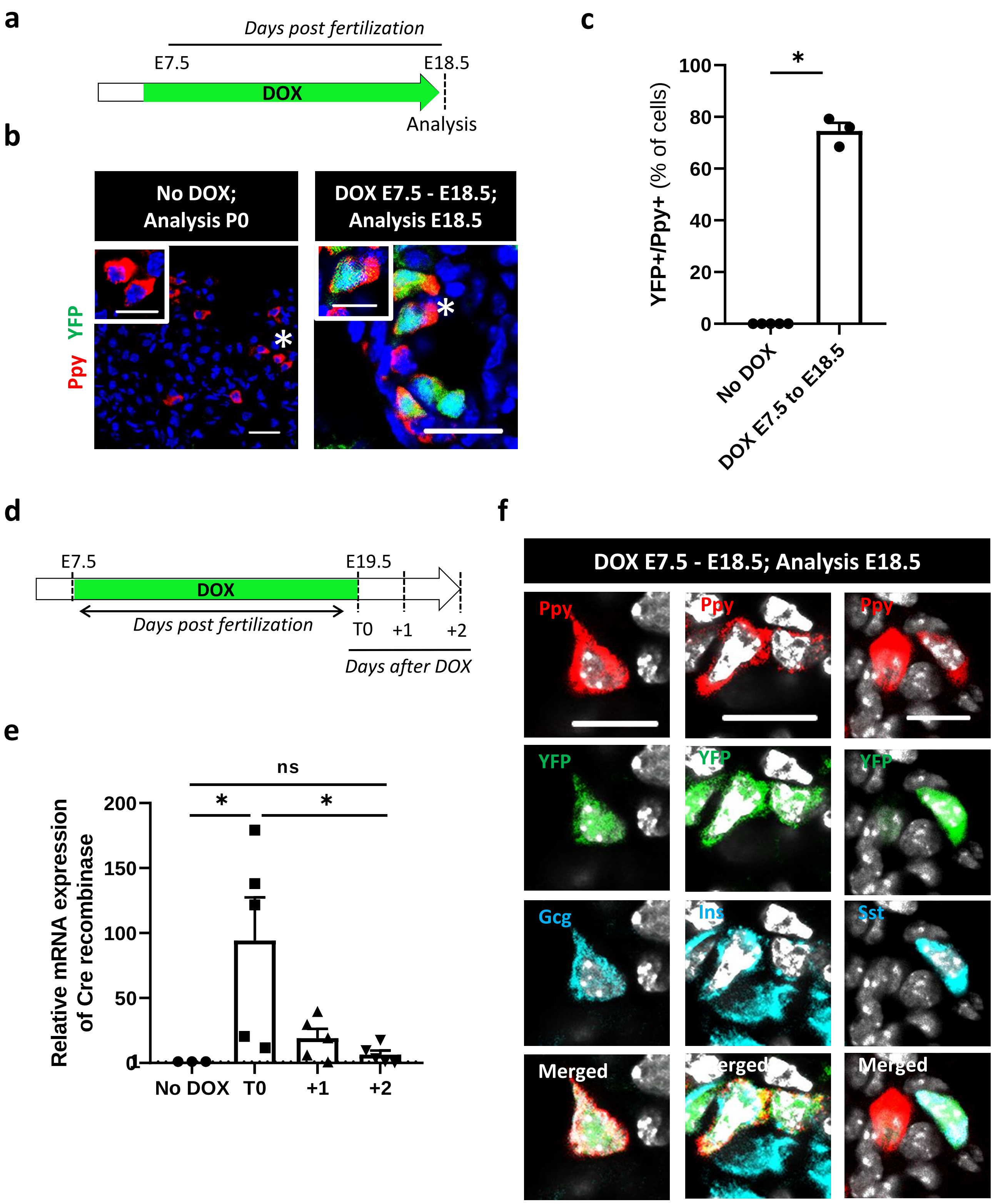
a
Days post fertilization
E7.5
E18.5
DOX
Analysis
b
No DOX; Analysis P0
DOX E7.5 - E18.5; Analysis E18.5
YFP
Ppy
c
YFP+/Ppy+ (% of cells)
100
80
60
40
20
0
*
No DOX
DOX E7.5 to E18.5
d
E7.5
E19.5
DOX
Days post fertilization
T0
+1
+2
Days after DOX
e
Relative mRNA expression of Cre recombinase
200
150
100
50
1
ns
*
*
No DOX
T0
+1
+2
f
DOX E7.5 - E18.5; Analysis E18.5
Ppy
YFP
Gcg
Ins
Sst
Merged

Supplementary Figure 4. **The Ppy-YFPi model is suitable for studying the adult Ppy-cell origin in embryonic stages. a,** Experimental design. **b,** Representative pictures of No DOX (control) and DOX E7.5-E18.5. Immunofluorescence: Ppy in red and YFP in green. **c,** Proportion of $Ppy^{+}$ cells YFP-labelled. YFP-traced $\gamma$-cells are only present upon DOX administration. No DOX (n=5 mice; 0 Ppy+YFP+ out of 2515 Ppy+ cells scored) and DOX E7.5 to E18.5 (n=3 mice, 378 Ppy+YFP+ out of 492 Ppy+ cells scored). Two-tailed Mann-Whitney test, No DOX vs DOX E7.5 to E18.5 P value = 0.0179. **d,** Experimental protocol. DOX administration from E7.5 to E19.5 to pregnant females (T0: under DOX exposure, +1: one day after DOX removal, +2: 2 days after DOX removal). **e,** Relative mRNA expression of *Cre* recombinase normalized on Ppy expression at different DOX administration timings (no DOX, n=3 mice; T0, n=5 mice; +1, n=5 mice; +2, n=5 mice). Data are shown as fold change of normalized ct values relative to No DOX (No DOX = 1). Two-tailed Mann-Whitney test (ns $p>0.05$; * $p\leq0.05$; ** $p\leq0.01$; *** $p\leq0.001$), No DOX versus T0: *P* value = 0.0357, T0 versus +2: *P* value = 0.0159, No DOX versus +2: *P value* = 0.25. **f.** Bihormonal YFP-labelled Ppy-expressing cells were detected at E18.5 (Ppy, red; YFP, green and Gcg, cyan). Scale bars: 20μm (10μm in insets). Data is presented as mean values ± s.e.m. Region of the pancreas: whole pancreas. Source data are provided as Source Data file (table d).

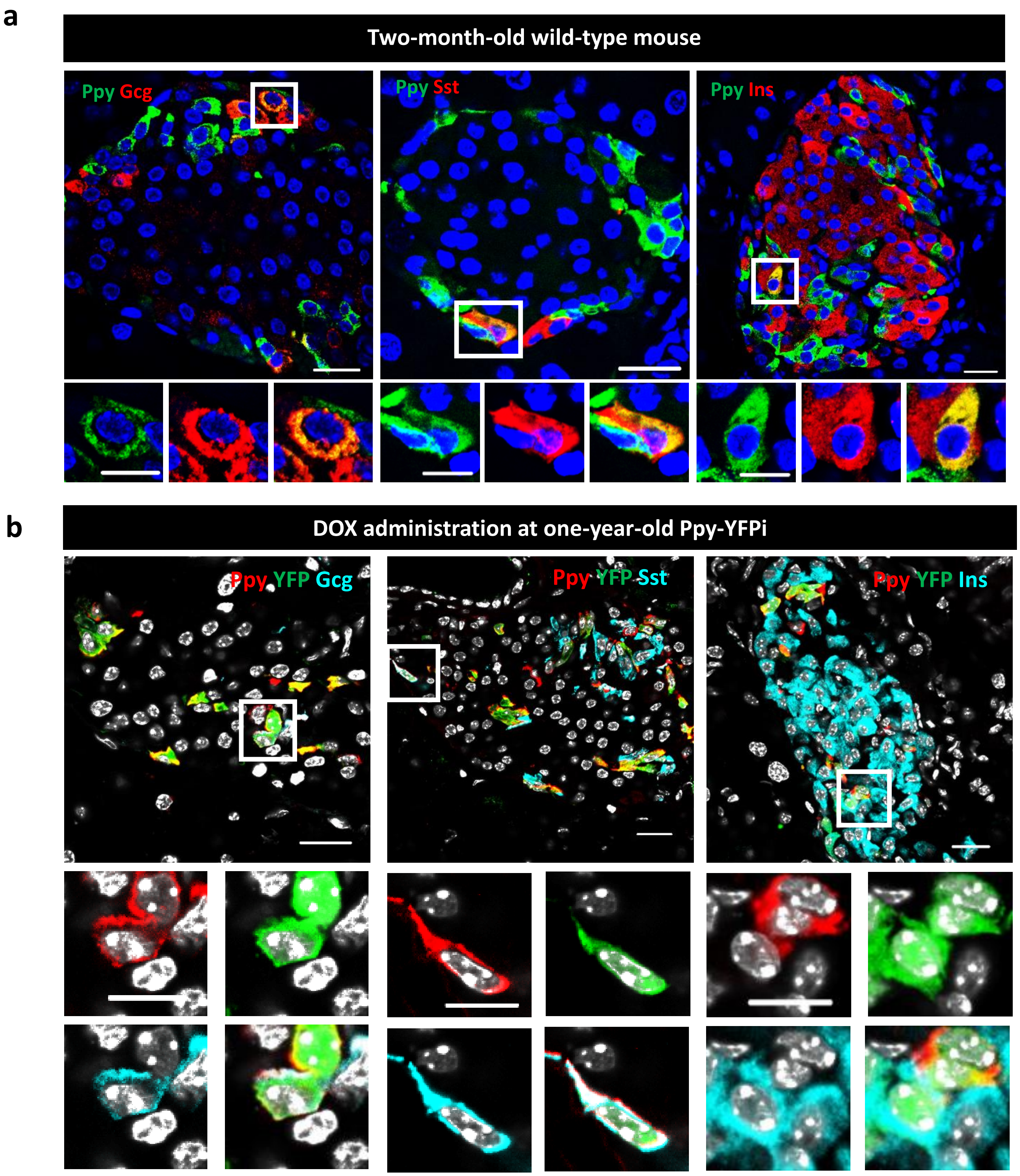


Supplementary Figure 5. **γ-cell heterogeneity in wild-type mice persist throughout life. a,** Bihormonal $Ppy^+Gcg^+$ (left; Ppy: green and Gcg: red), $Ppy^+Sst^+$ (middle; Ppy: green and Sst: red) and $Ppy^+Ins^+$ (right; Ppy: green and Ins: red) were detected in 2-month-old wild-type mice (n=5 mice). **b,** Heterogeneity in γ-cell population is maintained in one-year-old Ppy-YFPi mice (n=4 mice). Bihormonal $Ppy^+Gcg^+$ (left; Ppy: red and Gcg: cyan), $Ppy^+Sst^+$ (middle; Ppy: red and Sst: cyan) and $Ppy^+Ins^+$ (right; Ppy: red and Ins: cyan) YFP (green)-labelled could be identified. Scale bars: 20μm (10μm in insets). Region of the pancreas: Ventral. Source data are provided as Source Data file (table f).

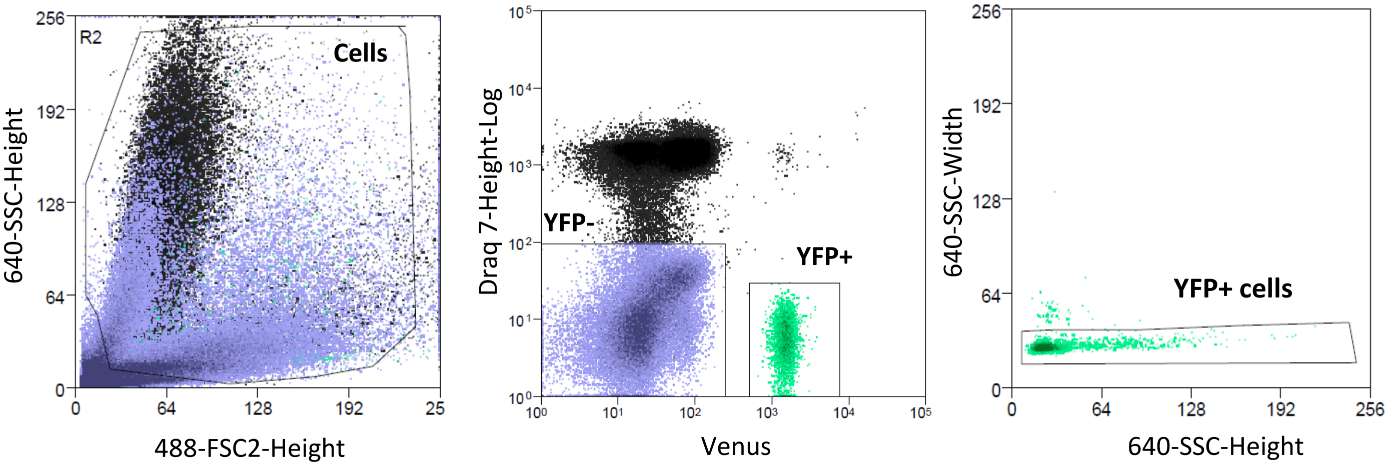


Supplementary Figure 6. **Gating strategy used for FACS sorting the YFP-labelled Ppy-expressing cells for RNA-seq analysis.** Representative FACS plots for Fig. 4-5.

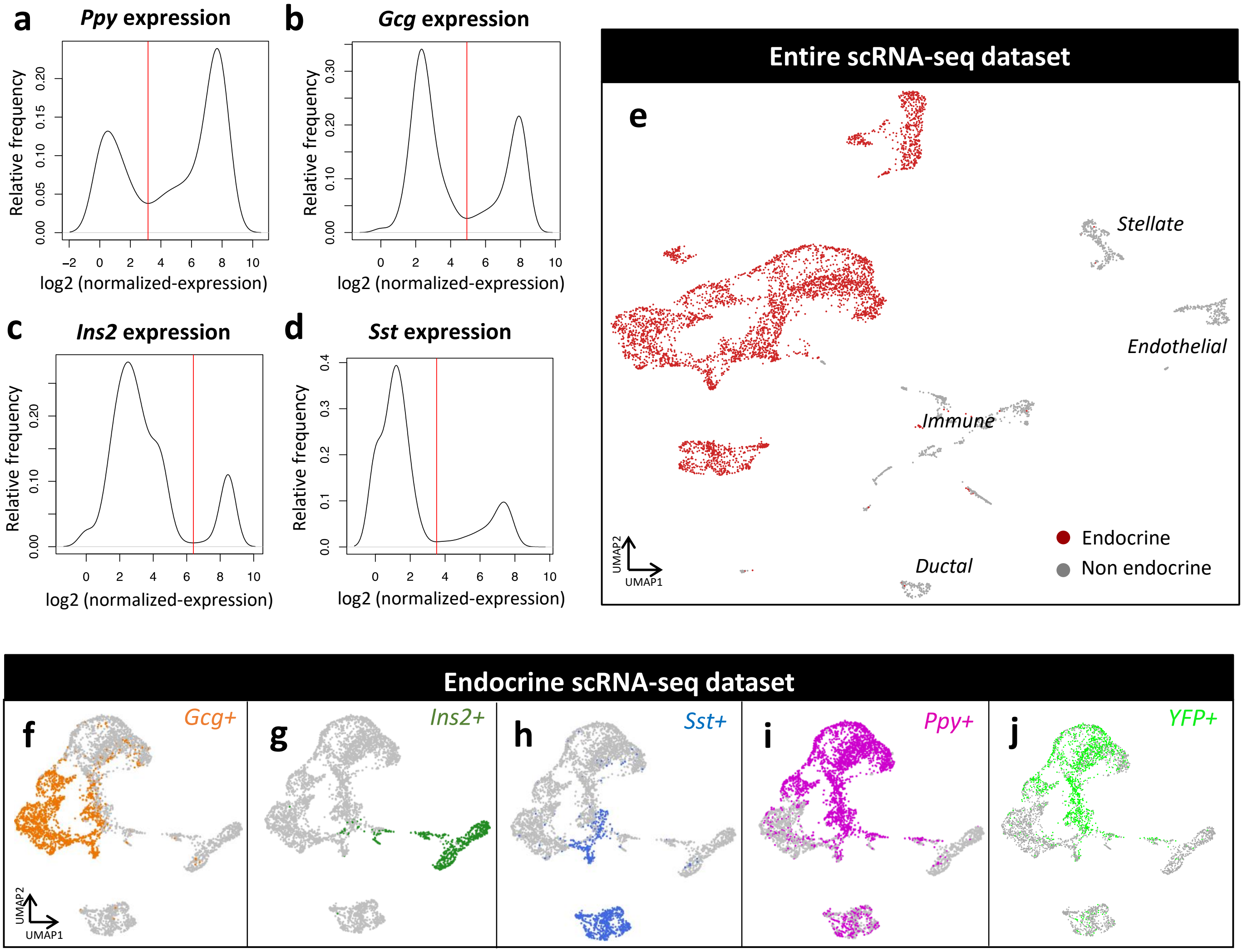


Supplementary Figure 7. **Single-cell transcriptomics of Ppy-YFPi islets. a-d,** Density plots of *Ppy* **(a)**, *Gcg* **(b)**, *Ins2* **(c)** and *Sst* **(d)** expression using normalized data from Ppy-YFPi islets. Red line indicates the threshold to define hormone+ cells. **e,** UMAP representation of all cells included in the Ppy-YFPi dataset (n=4261 cells). Red dots illustrate the endocrine population, grey dots represent all non-endocrine populations. **f-j**, UMAP representation of hormone positive cells: *Gcg*$^{+}$ (orange, n=1367 cells, **f**), *Ins2*$^{+}$ (green, n=548 cells, **g**), *Sst*$^{+}$ (blue, n=624 cells, **h**) and *Ppy*$^{+}$ (magenta, n=2626 cells, **i**) and *YFP*+ (fluor-green, n=1537 cells, **j**). Source data are provided as Source Data file (table g).

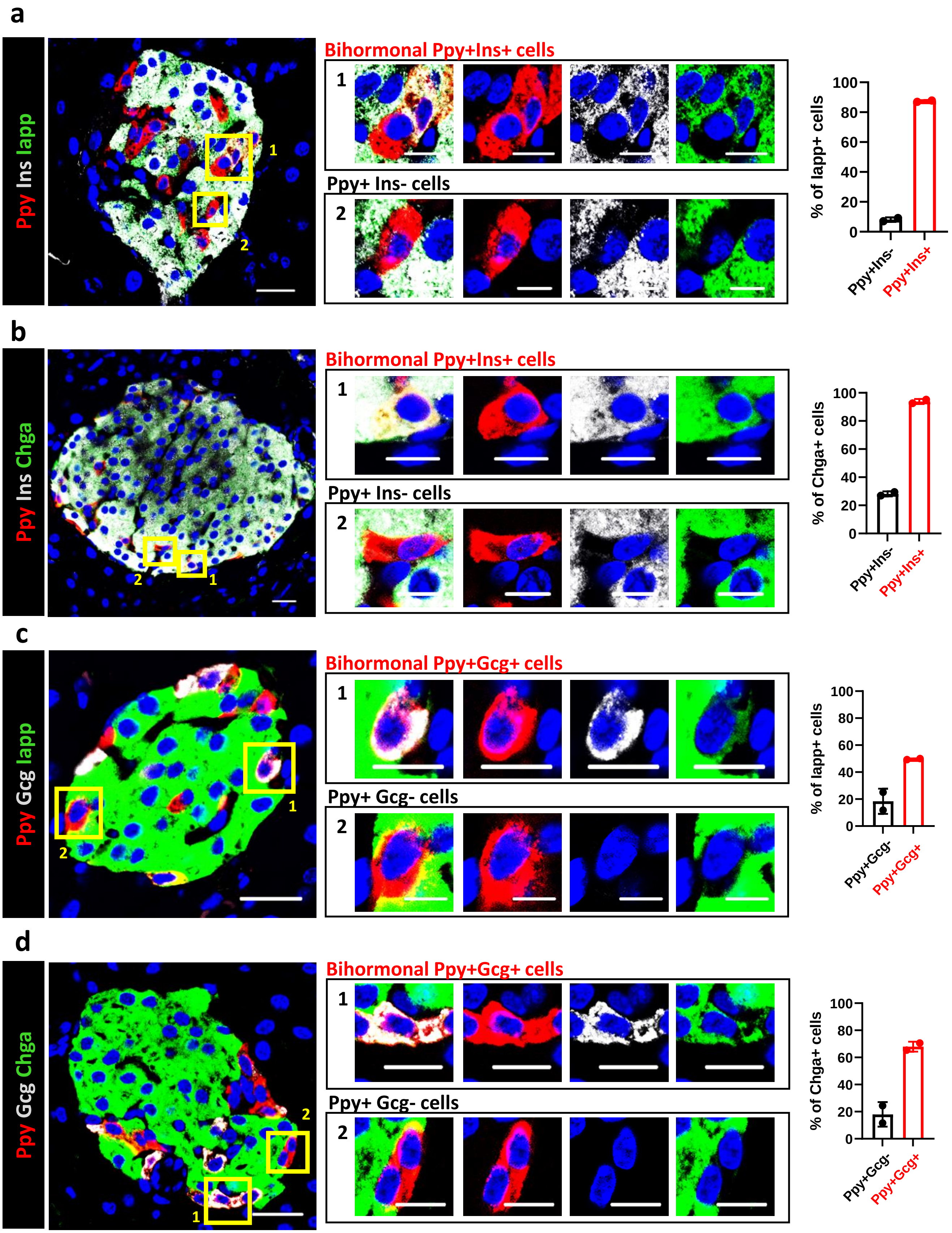

a
Ppy Ins Iapp
1
2
Bihormonal Ppy+Ins+ cells
Ppy+ Ins- cells
% of Iapp+ cells
100
80
60
40
20
0
Ppy+Ins-
Ppy+Ins+
b
Ppy Ins Chga
Bihormonal Ppy+Ins+ cells
Ppy+ Ins- cells
% of Chga+ cells
Ppy+Ins-
Ppy+Ins+
c
Ppy Gcg Iapp
Bihormonal Ppy+Gcg+ cells
Ppy+ Gcg- cells
% of Iapp+ cells
Ppy+Gcg-
Ppy+Gcg+
d
Ppy Gcg Chga
Bihormonal Ppy+Gcg+ cells
Ppy+ Gcg- cells
% of Chga+ cells
Ppy+Gcg-
Ppy+Gcg+

Supplementary Figure 8. **Protein validation of the bihormonal Ppy-expressing cell identity. a-d,** An enrichment in the production of Chga (**b, d**) and Iapp (**a, c**) was observed and quantified in bihormonal Ppy+Ins+ (**a, b**) and Ppy+Gcg+ (**c, d**) in wild-type mice (n=2 mice). Scale bar: 20μm (islet) or 10μm (cell). Percentage of Chga+ (**b, d; right pannel**) and Iapp+ (**a, c; right pannel**) cells in bihormonal Ppy+Ins+ and Ppy+Gcg+ compared to Ppy+Ins- and Ppy+Gcg- cells, respectively.
49 out of the 56 and 64 out of 68 Ppy+Ins+ cells expressed Iapp and Chga, respectively; compared to the 50 out of 617 and 234 out of 839 Ppy+Ins- cells that express Iapp and Chga, respectively. Similarly, 181 out of the 365 and 133 out of 198 Ppy+Gcg+ cells expressed Iapp and Chga, respectively; compared to the 122 out of 671 and 87 out of 520 Ppy+Gcg- cells that express Iapp and Chga, respectively. Ppy (a-d): red, Ins (a,b)/Gcg (c,d): grey and Iapp (a,c)/ChgA (b,d): green. Region of the pancreas: Ventral. Data is presented as mean values ± s.d. See Source data are provided as Source Data file (table m).

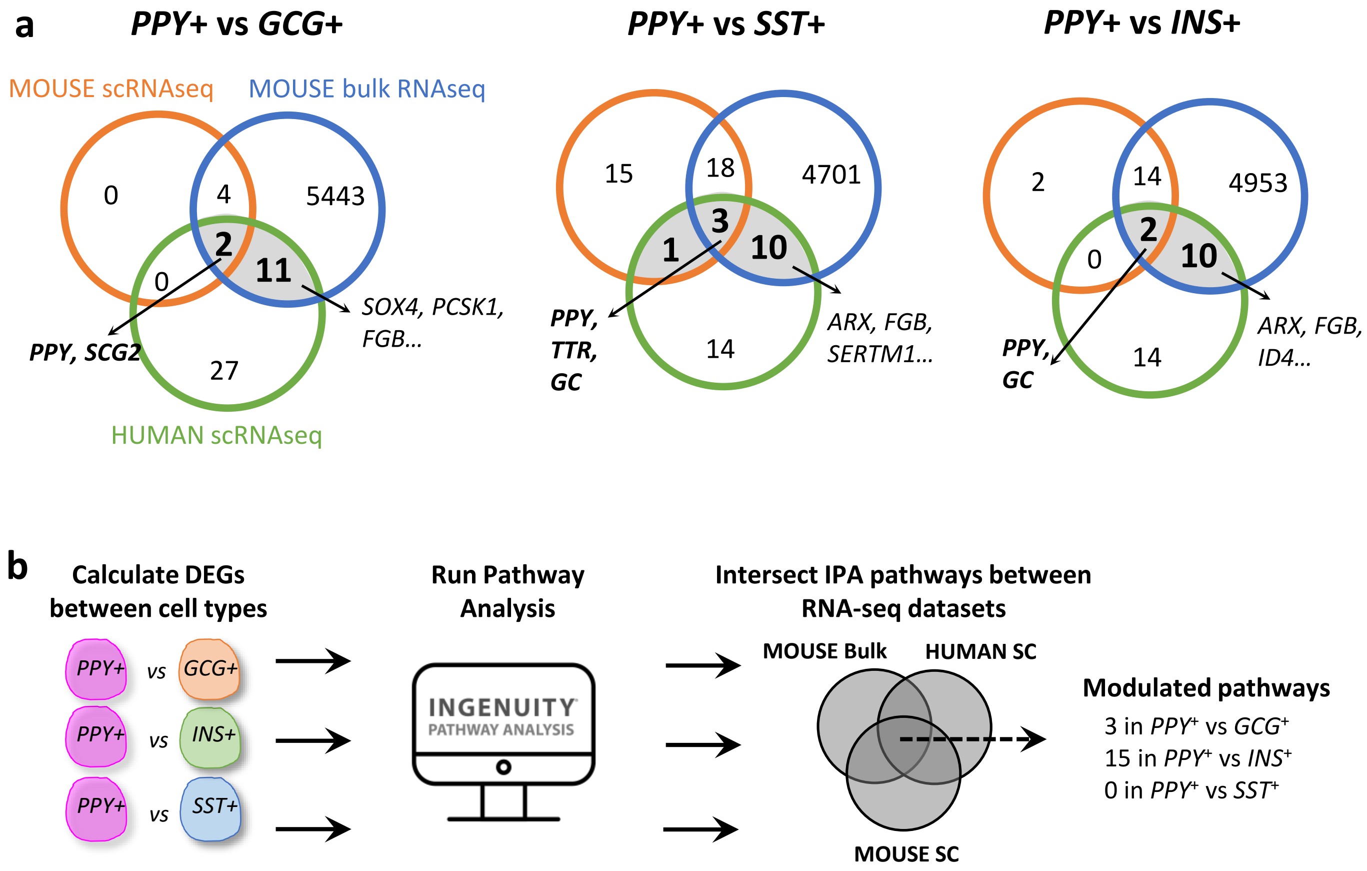


**c** ***PPY+ vs GCG+***

| Modulated Pathway (3) / State | |
|---|---|
| Role of PKR in Interferon Induction and Antiviral Response | Activated |
| Aryl Hydrocarbon Receptor Signaling | Activated |
| PI3K/AKT Signaling | Inhibited |

**d** ***PPY+ vs INS+***

| Modulated Pathway (15) / State | |
|---|---|
| Necroptosis Signaling Pathway | Inhibited |
| RhoA Signaling | Inhibited |
| Regulation of Actin-based Motility by Rho | Inhibited |
| Remodeling of Epithelial Adherens Junctions | Inhibited |
| ILK Signaling | Inhibited |
| PI3K/AKT Signaling | Inhibited |
| Aryl Hydrocarbon Receptor Signaling | Inhibited |
| Protein Kinase A Signaling | Inhibited |
| AMPK Signaling | Inhibited |
| Myc Mediated Apoptosis Signaling | Inhibited |
| p53 Signaling | Inhibited |
| Cardiac β-adrenergic Signaling | Inhibited |
| BAG2 Signaling Pathway | Inhibited |
| PPARα/RXRα Activation | Activated |
| PPAR Signaling | Activated |

Supplementary Figure 9. **Common transcriptomic identities between mouse and human γ-cells. a,** Common DEGs between PPY+ cells and each of the other endocrine cells in mouse and human. **b,** Experimental workflow to define γ-ID pathways using the mouse and human RNA-seq datasets. **c-d,** 3 and 15 differentially modulated pathways in γ-cells were identified when compared to α-(**c**) and β-cells (**d**), respectively, in both the murine and the human RNA-seq datasets. No commonly modulated pathway were identified in the Ppy cell population compared to and δ-cells (**b**) between mice and humans. The top modulated pathways are ranked based on Z-score. Activated pathways (orange), inhibited pathways (blue). Source data are provided as Source Data file (table o, p).

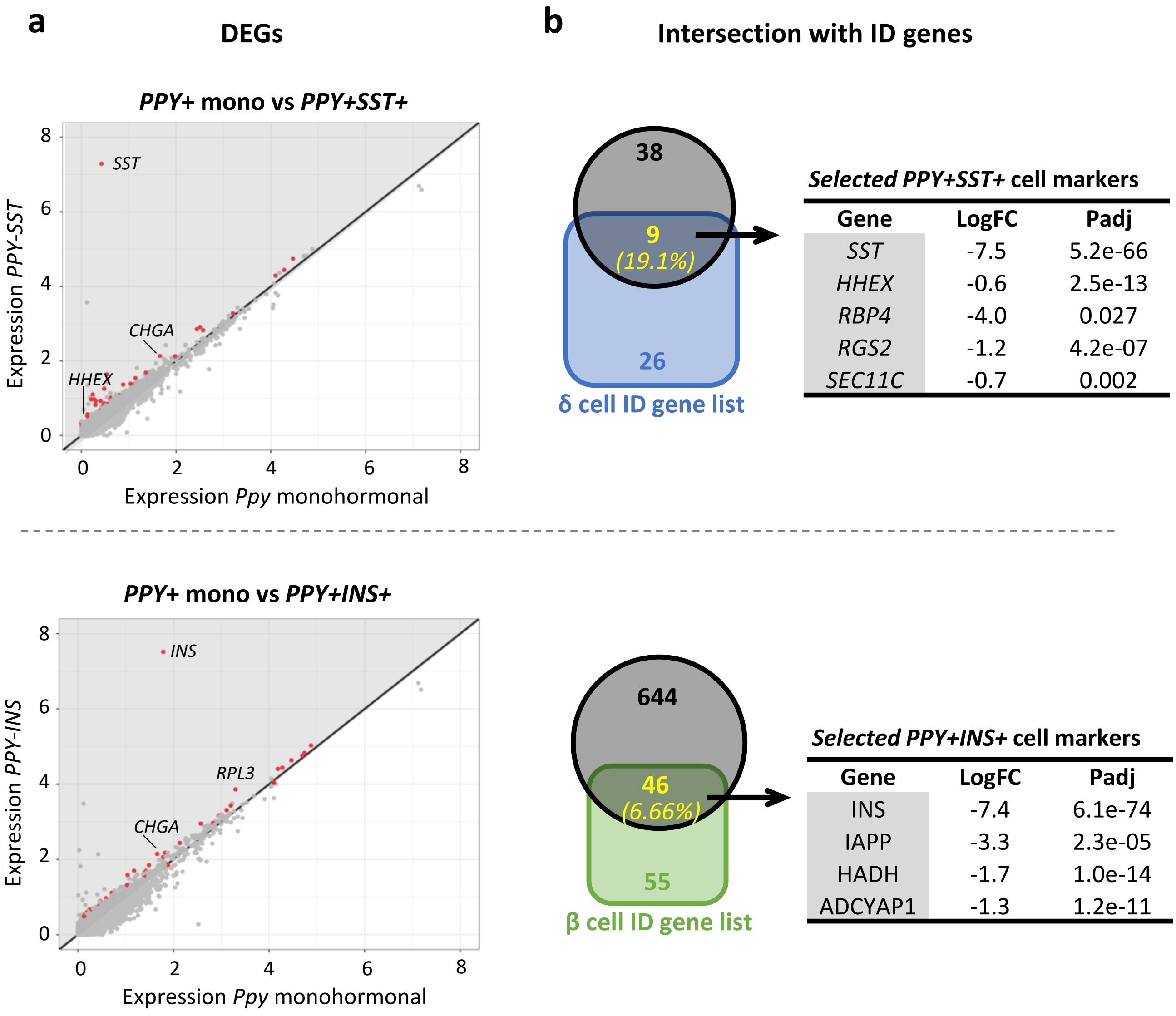


Supplementary Figure 10. **Transcriptomic profile of human bihormonal *PPY-SST* and *PPY-INS* cells. a,** Expression plot of differentially expressed genes (DEGs) between bihormonal *PPY-SST* (top) and *PPY-INS* (bottom) compared to monohormonal PPY-expressing cells in human. Each dot represents one gene. Black dots represent the genes with a *P value* >0.05, red dots represent the genes with *P value <0.05*. Gene names label the top DEGs. Grey area indicates the upregulated genes in each bihormonal population. Differential expression was calculated using a negative binomial generalized linear model in a pairwise manner between populations, taking along the number of UMIs and the number of genes as variables to regress. *P*-values (in panel **b** and Source Data s) are Bonferroni corrected based on the total number of genes in each dataset. **b,** Intersection of the upregulated genes in $PPY^{+}SST^{+}$ (top) and $PPY^{+}INS^{+}$ (bottom) from **a**, with human δ- and β-cell ID gene lists. All bihormonal *PPY*-expressing cell types share important markers with adult δ-cells (9 out of 47) and β-cells (46 out of 690). Source data are provided as Source Data file (table s).

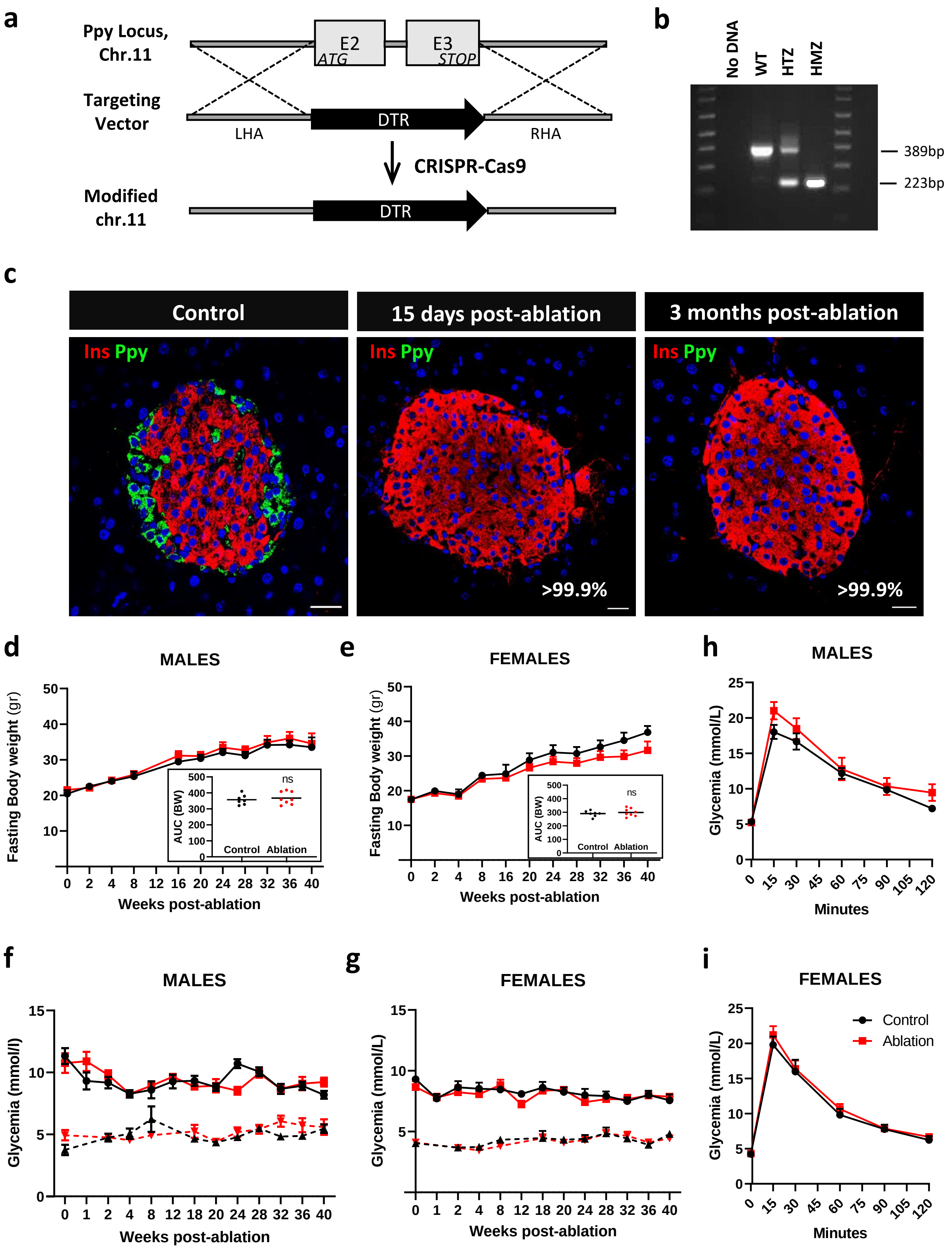
a
Ppy Locus, Chr.11
E2
ATG
E3
STOP
Targeting Vector
DTR
LHA
RHA
CRISPR-Cas9
Modified chr.11
DTR
b
No DNA
WT
HTZ
HMZ
389bp
223bp
c
Control
15 days post-ablation
3 months post-ablation
Ins Ppy
Ins Ppy
Ins Ppy
>99.9%
>99.9%
d
MALES
Fasting Body weight (gr)
AUC (BW)
ns
Control Ablation
Weeks post-ablation
e
FEMALES
Fasting Body weight (gr)
AUC (BW)
ns
Control Ablation
Weeks post-ablation
h
MALES
Glycemia (mmol/L)
Minutes
f
MALES
Glycemia (mmol/l)
Weeks post-ablation
g
FEMALES
Glycemia (mmol/L)
Weeks post-ablation
i
FEMALES
Control
Ablation
Glycemia (mmol/L)
Minutes

Supplementary Figure 11. **Ppy-expressing γ-cell ablation has no impact on body weight and glycemia**. **a,** CRISPR-Cas9 strategy to replace the Ppy coding sequence from chromosome 11 by the DTR (diphtheria toxin receptor) sequence. LHA, left homology arm; RHA, right homology arm. **b,** PCR products of wild-type (WT), Ppy-DTR heterozygous (HTZ) and Ppy-DTR homozygous (HMZ) mice (WT band: 389bp; KI band: 223bp). No DNA corresponds to the negative control. In all subsequent experiments, only Ppy-DTR heterozygous (HTZ) were used. **c,** Ppy-expressing cells were efficiently ablated 15 and 3 months post-DT injection (Ppy: green; Ins: red). Region of the pancreas: Ventral. Scale bar: 20μm. No DT (n=9 mice, 3656 Ppy+ cells scored), 15 days post-ablation (n=7 mice, 2 Ppy+ cells scored) and 3 months post-ablation (n=5 mice, 7 Ppy+ cells scored). **d-e**, Fasting body weight curves in non-ablated Ppy-DTR controls (black) and Ppy-cell ablated (red) males **(d)** and females **(e)** mice. Area under de curve (AUC) of each genotype is shown in the bottom right panel. **f-g,** Glycemia curves in non-ablated Ppy-DTR controls (black) and Ppy-cell ablated (red) males **(f)** and females **(g)** mice. Solid line represents random fed glycemia values, dashed line represents fasting glycemia values. Body weight and glycemia values were obtained from the same mice. Males: controls, n=7 mice; ablated, n=8 mice. Females: controls, n=9 mice; ablated, n=12 mice. **h-i**, Intraperitoneal glucose tolerance test to six months post-ablation Ppy-DTR mice (red) compared to non-injected controls (black) males **(e)** and females **(f)** mice. Males: controls, n=7 mice; ablated, n=7 mice. Females: controls, n=8 mice; ablated, n=12 mice. Data is presented as mean values ± s.e.m. Two-tailed Mann-Whitney test (ns p>0.05; * p≤0.05; ** p≤0.01; *** p≤0.001). Source data are provided as Source Data file (table v).

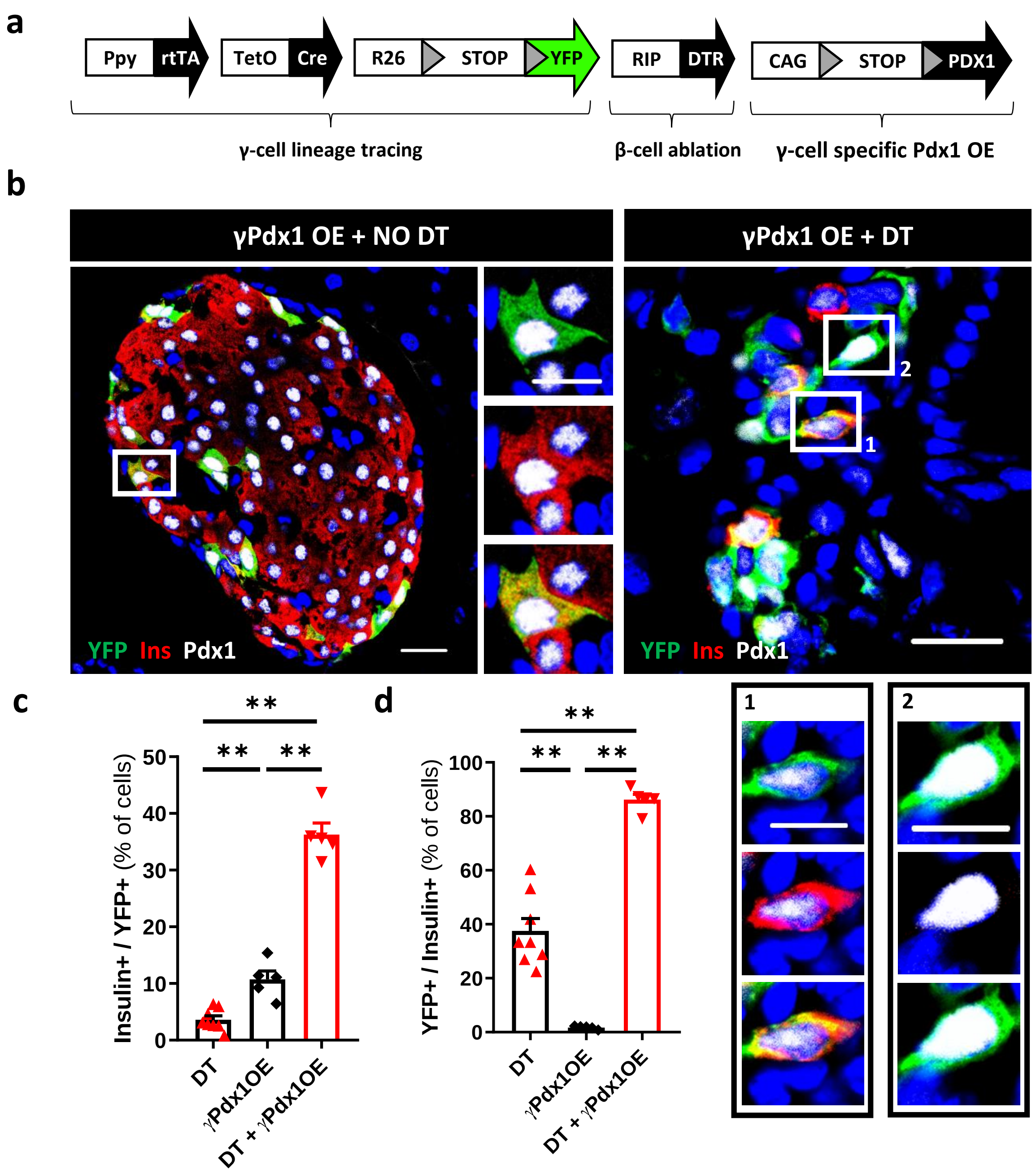
a
Ppy
rtTA
TetO
Cre
R26
STOP
YFP
RIP
DTR
CAG
STOP
PDX1
γ-cell lineage tracing
β-cell ablation
γ-cell specific Pdx1 OE
b
γPdx1 OE + NO DT
γPdx1 OE + DT
YFP Ins Pdx1
YFP Ins Pdx1
2
1
c
Insulin+ / YFP+ (% of cells)
DT
γPdx1OE
DT + γPdx1OE
d
YFP+ / Insulin+ (% of cells)
DT
γPdx1OE
DT + γPdx1OE
1
2

Supplementary Figure 12. **Synergistic γ-cell reprogramming by inducing Pdx1 expression and ablating β-cells. a,** Transgenes required for γ-cell lineage tracing and ectopic Pdx1 expression combined with β-cell ablation. **b,** YFP-labelled γ-cells express Pdx1 in control and DT-treated mice. Approximately, half of the γ-cells expressing Pdx1 engage insulin production after β-cell ablation. The experiment was performed once with mice treated asynchronously depending on their availability. Scale bar: 20μm (islet) or 10μm (cell). Insulin: red; YFP: green; Pdx1: white. **c,** Percentage of $YFP^+$ γ-cells expressing insulin in γPdx1 after β-cell loss. Two-tailed Mann-Whitney test (ns $p>0.05$; * $p\leq0.05$; ** $p\leq0.01$; *** $p\leq0.001$), DT versus γPdx1OE: *P* value = 0.0016, γPdx1OE versus DT+γPdx1OE: *P* value = 0.0079, DT versus DT+γPdx1OE: *P* value = 0.0016. **d,** Percentage of Insulin+ cells labelled with YFP in γPdx1 after β-cell loss. Two-tailed Mann-Whitney test, DT versus γPdx1OE: *P* value = 0.0016, γPdx1OE versus DT+γPdx1OE: *P* value = 0.0079, DT versus DT+γPdx1OE: *P* value = 0.0016. DT, n=8 mice, 209 Ins+YFP+ out of 5477 YFP+ cells scored (this condition is taken for reference from Fig. 8e-f); γPdx1, n=5 mice, 233 Ins+YFP+ out of 2301 YFP+ cells scored; γPdx1+DT, n=5 mice, 652 Ins+YFP+ out of 1784 YFP+ cells scored. Region of the pancreas: Ventral. Data is presented as mean values ± s.e.m. Source data are provided as Source Data file (table x).

**Supplementary Table 1**

List of primer sequences for qPCR

| ***Gene*** | **Primer sequence forward** | **Primer sequence reverse** |
|---|---|---|
| *B-actin* | 5' AAGGCCAACCGTGAAAAGAT 3' | 5' GTGGTACGACCAGAGGGATAC 3' |
| *Gapdh* | 5’ TCCATGACAACTTTGGCATTG 3’ | 5’ CAGTCTTCTGGGTGGCAGTGA 3’ |
| *Ppy* | 5' GAGCTGAAGGCAGGGAGC 3' | 5' GTTTGCAAGGGAGCAGGTTG 3' |
| *Cre* | 5' GCCGCGCGAGATATGG 3' | 5' GCCACCAGCTTGCATGATC 3' |
| *GFP* | 5' CTACCCCGACCACATGAAGC 3' | 5' GTAGTTGCCGTCGTCCTTGA 3' |
| *Pyy* | 5' CGCAGCTCTGTTCTCCAAC 3' | 5' CAAACCTTCTGGCCGAGACC 3' |

**Supplementary Table 2**

List of antibodies used for immunofluorescence

| Primary antibodies | Dilution | Company |
|---|---|---|
| guinea pig anti-Pdx1 | 1/750 | C.W. Wright |
| guinea pig anti-porcine insulin | 1/400 | DAKO (A0564) |
| rabbit anti-insulin | 1/3000 | Molecular Probes (701265) |
| mouse anti-glucagon | 1/1000 | Sigma (G2654) |
| rabbit anti-glucagon | 1/200 | DAKO (A0565) |
| mouse anti-somatostatin | 1/200 | BCBC Ab1985 |
| rabbit anti-somatostatin | 1/200 | DAKO (A0566) |
| goat anti-somatostatin* | 1/200 | Santa Cruz Biotechnology (sc-55565)* |
| rabbit anti-GFP | 1/400 | Molecular Probes (A11122) |
| chicken anti-GFP | 1/500 | Abcam (ab13970) |
| mouse anti-Ppy | 1/200 | Y. Fujitani |
| mouse anti-Ppy | 1/1000 | R&D Biosystems (MAB62971) |
| mouse anti-Pyy | 1/1000 | Abcam (ab112474) |
| rabbit anti-Chga | 1/200 | Abcam (ab68271) |
| rabbit anti-Iapp | 1/500 | Abcam (ab254259) |

** Out of stock*

| Secondary antibodies | Dilution | Company |
|---|---|---|
| Alexa 568 | 1/500 | Molecular Probes |
| Alexa 488 | 1/500 | Molecular Probes |
| Alexa 647 | 1/500 | Molecular Probes |
| Alexa 405 | 1/500 | Molecular Probes |
| Cy3 | 1/500 | Southern Biotech |
| Cy5 | 1/500 | Southern Biotech |
| FITC | 1/500 | Southern Biotech |
| TRITC | 1/500 | Southern Biotech |
