# Supplementary material for: Pancreatic Ppy-expressing γ-cells display mixed phenotypic traits and the adaptive plasticity to engage insulin production
Source: Nat Commun. 2021 Jul 22;12:4458. doi: 10.1038/s41467-021-24788-0 (PMC8298494; doi:10.1038/s41467-021-24788-0)
Supplement: Supplementary file 3 — Reporting Summary [file 41467_2021_24788_MOESM3_ESM.pdf]

nature research

Corresponding author(s): Pedro Luis Herrera

Last updated by author(s): May 28, 2021

Reporting Summary

Nature Research wishes to improve the reproducibility of the work that we publish. This form provides structure for consistency and transparency in reporting. For further information on Nature Research policies, see our Editorial Policies and the Editorial Policy Checklist.

Statistics

For all statistical analyses, confirm that the following items are present in the figure legend, table legend, main text, or Methods section.

n/a	Confirmed	
☐	☒	The exact sample size (*n*) for each experimental group/condition, given as a discrete number and unit of measurement
☐	☒	A statement on whether measurements were taken from distinct samples or whether the same sample was measured repeatedly
☐	☒	The statistical test(s) used AND whether they are one- or two-sided<br>*Only common tests should be described solely by name; describe more complex techniques in the Methods section.*
☐	☒	A description of all covariates tested
☒	☐	A description of any assumptions or corrections, such as tests of normality and adjustment for multiple comparisons
☐	☒	A full description of the statistical parameters including central tendency (e.g. means) or other basic estimates (e.g. regression coefficient) AND variation (e.g. standard deviation) or associated estimates of uncertainty (e.g. confidence intervals)
☐	☒	For null hypothesis testing, the test statistic (e.g. *F*, *t*, *r*) with confidence intervals, effect sizes, degrees of freedom and *P* value noted<br>*Give P values as exact values whenever suitable.*
☒	☐	For Bayesian analysis, information on the choice of priors and Markov chain Monte Carlo settings
☒	☐	For hierarchical and complex designs, identification of the appropriate level for tests and full reporting of outcomes
☒	☐	Estimates of effect sizes (e.g. Cohen's *d*, Pearson's *r*), indicating how they were calculated

Our web collection on statistics for biologists contains articles on many of the points above.

Software and code

Policy information about availability of computer code

Data collection	CSDiva v 8.0.1 (BD Biosciences) for sorting on a FACSAria2. Summit v6.2 (Beckman Coulter) for sorting on a Moflo Astrios.
Data analysis	Single cell RNA-sequencing libraries were sequenced as 100bp paired-end reads on a HiSeq 40000 platform (Illumina). Demultiplex of barcodes was performed using Cell Ranger software pipeline (v2.0.1; 10x Genomics) using Ensembl mm10 build 84 reference genome. Data were loaded as Seurat objects (v3.1.0) under R (version 3.6.1) in R Studio (version 1.0.153) using the Read10X function. Data were normalized using the LogNormalize method, using 10'000 as scale factor. Data integration was performed using Seurat FindIntegrationAnchors function, using 20 CC's. Clustering was performed with a resolution of 0.1 after nearest neighbor detection. UMAP dimensional reduction was performed using the uwot method and the 30 nearest neighbors for local approximation. Differential expression between identities was calculated using the FindMarkers function in Seurat, using the negative binomial test on the RNA assay, with experiment replication set as a variable to regress, and a minimum log fold change set to 0.5.<br>Bulk RNA-sequencing libraries were prepared using Nextera XT DNA Sample Preparation kit (Illumina) and sequenced on an Illumina HiSeq 2500 or HiSeq4000 platform with single-end 100-bp reads or pair-end 100bp reads. Sequencing data was uploaded to, and aligned on the Galaxy project against Ensembl reference genome GRCm38.p6 (release 100) using STAR version 2.7.2b in 2-pass mapping mode. Aligned data were counted using HTSeq version 0.9.1 in union mode. Differential expression between identities was calculated using DESeq2 version 1.28.1 in Seurat. Pathway analysis was performed using the Ingenuity Pathway Analyses software (IPA) suit of tools v01-16 (Ingenuity Systems, Redwood City, CA).<br>Sections were examined with a confocal microscope (Leica TCS SPE).<br>In all experiments cells were manually counted and considered bihormonal or coexpressing markers when one nucleus was clearly surrounded by both hormone / reporter staining.<br>All statistical analyses were performed with GraphPad Prism8.0.

For manuscripts utilizing custom algorithms or software that are central to the research but not yet described in published literature, software must be made available to editors and reviewers. We strongly encourage code deposition in a community repository (e.g. GitHub). See the Nature Research guidelines for submitting code & software for further information.

Data

Policy information about availability of data

All manuscripts must include a data availability statement. This statement should provide the following information, where applicable:

- Accession codes, unique identifiers, or web links for publicly available datasets
- A list of figures that have associated raw data
- A description of any restrictions on data availability

The mouse scRNA-seq and the gamma- and delta-cell bulk RNA-seq dataset generated in this study have been deposited in the NCBI GEO database with the accession number GSE156665. The mouse bulk-RNA-seq of alpha- and beta-cells was obtained from the NCBI GEO database (accession number GSE155519). The human scRNA-seq data was obtained from the NCBI GEO database (accession number GSE150724).
All data and materials used are available from the authors or from commercially available sources.

Field-specific reporting

Please select the one below that is the best fit for your research. If you are not sure, read the appropriate sections before making your selection.

☒ Life sciences ☐ Behavioural & social sciences ☐ Ecological, evolutionary & environmental sciences

For a reference copy of the document with all sections, see nature.com/documents/nr-reporting-summary-flat.pdf

Life sciences study design

All studies must disclose on these points even when the disclosure is negative.

Sample size	Sample size was chosen to ensure adequate power and to detect a pre-specified effect based on the available literature and protocols in the field. Therefore, sample sizes are comparable with the ones used in the published literature in the field (Thorel, F. et al. 2010; Chera, S. et al. 2014; Cigliola, V. et al. 2018; Furuyama, K. et al. 2019).<br>For multiple transgenics and human samples the numbers were also limited by the availability of the phenotype.<br>No statistical methods were used to predetermine sample size.
Data exclusions	At the beginning of each experiment, mice must be (1) healthy, (2) normoglycemic, (4) bearing all the desired transgenes, (5) for age-matched controls we prefered, when possible, litter mates. These exclusion criteria were pre-established.
Replication	For all experiments, all attempts at replication were successful. All in vivo experiments were replicated at least once.
Randomization	In all experiments, we randomly selected in each litter the experimental and control animals.
Blinding	In vitro experiments, investigators were blinded to group allocation during data collection and analysis. But, no blinding was possible in the diphtheria toxin treatment experiments (induction of beta-cell ablation) due to regular glycemic control that must be done on these mice.

Reporting for specific materials, systems and methods

We require information from authors about some types of materials, experimental systems and methods used in many studies. Here, indicate whether each material, system or method listed is relevant to your study. If you are not sure if a list item applies to your research, read the appropriate section before selecting a response.

Materials & experimental systems

n/a	Involved in the study
☐	☒ Antibodies
☒	☐ Eukaryotic cell lines
☒	☐ Palaeontology and archaeology
☐	☒ Animals and other organisms
☐	☒ Human research participants
☒	☐ Clinical data
☒	☐ Dual use research of concern

Methods

n/a	Involved in the study
☒	☐ ChIP-seq
☐	☒ Flow cytometry
☒	☐ MRI-based neuroimaging

Antibodies

Antibodies used	The primary antibodies used were: guinea pig anti-Pdx1 (1/750; C.W. Wright), guinea pig anti-porcine insulin (1/400; DAKO, A0564), rabbit anti-insulin (1/3000; Molecular Probes, 701265), mouse anti-glucagon (1/1000; Sigma, G2654), rabbit anti-glucagon (1/200; DAKO, A0565), mouse anti-somatostatin (1/200; BCBC Ab1985), rabbit anti-somatostatin (1/200; DAKO, A0566), goat anti-somatostatin (1/200; Santa Cruz Biotechnology, sc-55565), rabbit anti-GFP (1/400; Molecular Probes, A11122), chicken anti-GFP (1/500; Abcam, ab13970), mouse anti-Ppy (1/200; Y. Fujitani, 10.1507/endocrj.EJ18-0441), mouse anti-Ppy (1/1000; R&D Biosystems,

	MAB62971), mouse anti-Pyy (1/1000; Abcam, ab112474), rabbit anti-Chga (1/200; Abcam, ab68271) and rabbit anti-Iapp (1/500; Abcam, ab254259). Secondary antibodies were coupled to Alexa 488, 405, 568, 647 (1/500; Molecular Probes) or TRITC, FITC, Cy3 and Cy5 (1/500; Southern Biotech).
Validation	All antibodies used were validated by the respective commercial source in either murine or human tissue for application in IF, IHC-P or WB.<br>The specificity of the two Ppy antibodies used in this study were also validated by us in mouse tissue (Suppementary Fig. 2).

Animals and other organisms

Policy information about studies involving animals; ARRIVE guidelines recommended for reporting animal research

Laboratory animals	All mice used in this studies had a mixed background. 2-3 months old male and female mice were used for all experiments. Except for long-term following of Ppy-KO and Ppy-cell ablated animals, which were kept until one-year-old.
Wild animals	No wild animals were used in this study.
Field-collected samples	No field-collected samples were used in this study.
Ethics oversight	The study follows all ethical regulations regarding animal experimentation, all experiments were performed under the guidelines of the Direction General de la Santé du Canton de Genève (license numbers: GE/111/17 and GE/121/17).

Note that full information on the approval of the study protocol must also be provided in the manuscript.

Human research participants

Policy information about studies involving human research participants

Population characteristics	Donor details for samples obtained through nPOD are available from the corresponding author upon reasonable request due to donor privacy.
Recruitment	ancreatic histologic samples were obtained from anonymized deceased patients through the nPOD (Network for Pancreatic Organ Donors with Diabetes), supported by JDRF (Juvenile Diabetes Research Foundation International) at the U. of Florida. Donor information and consent from the donor family were obtained for all nPOD samples.
Ethics oversight	All studies involving human samples were approved by ethical committee in University of Geneva.

Note that full information on the approval of the study protocol must also be provided in the manuscript.

Flow Cytometry

Plots

Confirm that:

- [x] The axis labels state the marker and fluorochrome used (e.g. CD4-FITC).
- [x] The axis scales are clearly visible. Include numbers along axes only for bottom left plot of group (a 'group' is an analysis of identical markers).
- [x] All plots are contour plots with outliers or pseudocolor plots.
- [x] A numerical value for number of cells or percentage (with statistics) is provided.

Methodology

Sample preparation	RNA-Seq analyses. Islet cells were obtained from two-month-old doxycycline or tamoxifen-treated Glucagon-rtTA; R26-YFP (Thorel et al 2010), SstCre; R26YFP (Chera et al 2014), RipCreER; R26YFP (Dor et al 2004) and Ppy-rtTA; R26-YFP mice. islet isolation and cell isolation by flow cytometry (FACS) were performed using previously described protocols (Chera et al 2014).
Instrument	Cells were sorted on a FACSAria2 (BD Biosciences) or Moflo Astrios (Beckman Coulter) system
Software	FACSDiva v 8.0.1 (BD Biosciences) for sorting on a FACSAria2. Summit v 6.2 (Beckman Coulter) for sorting on a Moflo Astrios. Kaluza Analysis v 2.0 (Beckman Coulter) for analysis.
Cell population abundance	For validation of the purity, small fractions of sorted cells were FACS-sorted again to confirm the gating strategy, and also evaluated by immunostaining, showing more than 99% abundance within the post-sort fractions.
Gating strategy	Single viable islet cells were gated by forward scatter, side scatter and pulse-width parameters and by negative staining for DAPI (Life Technologies) or DRAQ7 (B25595, BD Biosciences) to remove doublets and dead cells. Boundaries between positive and negative were very clear because of very high expression of reporter proteins.

- [x] Tick this box to confirm that a figure exemplifying the gating strategy is provided in the Supplementary Information.
